# The healthy user and healthy adherer bias: a nested case-control study among statin users in the Rotterdam Study

**DOI:** 10.1186/2049-3258-73-S1-O6

**Published:** 2015-09-17

**Authors:** Loes Hollestein, Ömer Baser, BHCh Stricker, T Nijsten

**Affiliations:** 1 Erasmus MC University Medical Center, Rotterdam, the Netherlands

## Objective

Failure to correct for lifestyle in routinely collected data may lead to the healthy user or healthy adherer bias, because patients who initiate or adhere to preventive therapy (e.g. statins) may have a healthier lifestyle than patients who do not use or adhere to statins, respectively. We aimed to assess if there is a difference in lifestyle between statin users and non-users and between adherent and nonadherent statin users.

## Research design and methods

We conducted a nested case-control study within the Rotterdam Study. In order to examine the healthy user bias, statin initiators were matched to 10 controls on age, sex and follow-up time. All statin users with at least 5 years follow-up were included in order to examine the healthy adherer bias. Subjects with a medication possession ratio (MPR) of = 80% during these 5 years were classified as adherent users and matched to 10 non-adherent statin users on age and sex. Univariable conditional logistic regression was used to compute odds ratios (OR) with 95% confidence intervals (CI).

## Results

We included 705 statin initiators and 7046 matched non-users. Smoking habits and physical activity were equal: OR current smoker vs never smoker 0.96 (95% CI: 0.79-1.17); OR frequent physical activity vs moderate physical activity 1.31 (95% CI: 0.79-2.16). Statin users were more likely to drink alcohol and have overweight: OR alcohol drinker vs nondrinkers: 1.36 (95% CI: 1.15-1.62), OR obesity vs normal weight: 1.15 (95% CI: 0.89-1.47). Statin users were more likely to have a healthy diet: OR healthy diet vs normal diet: 1.32 (95% CI: 1.05-1.67).

## Conclusion

Statin users do not engage in healthier behavior with regard to all lifestyle factors. The amount of residual confounding due to the healthy user bias in routinely collected data remains uncertain and may depend on the research question.

